# 

**Published:** 1891-01-10

**Authors:** 


"Undoubtedly many persons have been starved to death through inexperienced medical men and nurses placing an undue nutritious value on such preparations as Extract of Meat, home-made beef-tea, &c., whereas had JOHNSTON'S BOVRIL been used in their stead, the patients would in nine cases out of ten have gained strength to battle against the disease under which they were suffering. I have no hesitation to advise you [illegible]e to your patients, to your convalescents, to those of your clients who have mental exertions, to use JOHNSTON'[illegible] which, in a concentrated form, contains a substantial tonic and a palatable food."—J. M. BEAUSOLIEL, M.D.

SOLD BY

CHEMISTS, GROCERS. &c.

THROUGHOUT

THE UNITED KINGDOM.

### THE HOSPITAL

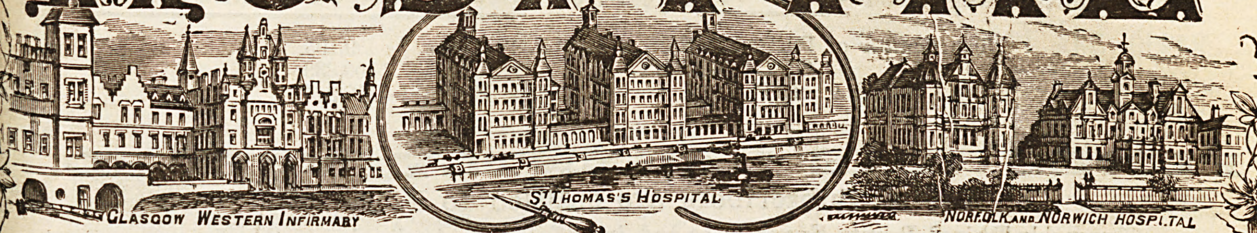

WITH EXTRA NURSING SUPPLEMENT.

[No. 224. Vol IX.] SATURDAY, JANUARY 10th, 1891. [illegible] ONE PENNY.

